# Online Mastermind Groups: A Non-hierarchical Mentorship Model for Professional Development

**DOI:** 10.7759/cureus.3013

**Published:** 2018-07-20

**Authors:** Glenn Paetow, Fareen Zaver, Michael Gottlieb, Teresa M Chan, Michelle Lin, Michael A Gisondi

**Affiliations:** 1 Emergency Medicine, Hennepin County Medical Center, Minneapolis, USA; 2 Emergency Medicine, University of Calgary, Calgary, CAN; 3 Emergency Medicine, Rush University Medical Center, Chicago, USA; 4 Faculty of Health Sciences, McMaster University, Hamilton, CAN; 5 Department of Emergency Medicine, UCSF School of Medicine, San Francisco, USA; 6 Emergency Medicine, Stanford University School of Medicine, Palo Alto, USA

**Keywords:** mastermind group, mastermind, mentorship, online mentorship, non-heirarchical, professional development, faculty development, mentor

## Abstract

Mentorship is an important driver of professional development and scholarship in academic medicine. Several mentorship models have been described in the medical education literature, with the majority featuring a hierarchical relationship between senior and junior members of an institution. ‘Mastermind Groups’, popularized in the business world, offer an alternative model of group mentorship that benefits from the combined intelligence and accumulated experience of the participants involved. We describe an online application of the Mastermind model, used as an opportunity for faculty development by a globally distributed team of health professions educators. The majority of our participants rated their experiences over two online Mastermind group mentoring sessions as ‘very valuable’, resulting in recommendations of specific developmental resources, professional referrals, and identifiable immediate ‘next steps’ for their careers. Our experience suggests that online Mastermind groups are an effective, feasible, zero-cost model for group mentorship and professional development in medicine.

## Introduction

Mentorship is an essential component of professional development and its benefits are well described in the medical, business, and education literature [1-10]. Mentorship relationships are beneficial for both mentors and mentees, who experience increased career satisfaction, scholarship, and efficiency of academic promotion [11-12]. Models include one-on-one mentor-protege models [13], institutional group programs [14-15], and “speed-mentoring” sessions with many mentors in serial succession [16].

Recently, a collaborative, network-based model for mentorship has gained popularity in the business world: the Mastermind group [17-20]. Initially described by Napoleon Hill [21], the Mastermind group is composed of multiple colleagues, including near-peers and those at different stages of their academic careers, who provide mentorship and career advice for each other through regularly scheduled meetings. The group benefits from the combined intelligence and accumulated experience of the participants.

Advances in technology now mean that the world can literally be right at our fingertips. Geographic bounding of the mentor-mentee relationship is no longer necessary [22]. In fact, for effective mentorship through Mastermind Groups, it may be advantageous to connect academic physicians from different centers in mentorship opportunities, since these relationships may be less influenced by personal gains and local politics.

We describe the curriculum design, implementation, and program evaluation of a Mastermind group for the purpose of professional development of academic emergency physicians. Our outcome measures reflect participant experience, impact, and feasibility of the program.

## Materials and methods

Participants

Academic Life in Emergency Medicine (ALiEM; www.aliem.com) conducted two Mastermind group sessions in 2017 for volunteer faculty within the organization. ALiEM is a digital health professions education organization with a globally distributed team of faculty who lead various online educational initiatives. The Mastermind group was intended as a career development exercise for the volunteer faculty in the organization. Participants were recruited from within ALiEM via the organizational Slack Channel (Slack Technologies Inc, San Francisco, CA) through a general message to all volunteer faculty at ALiEM, asking for voluntary participation in this faculty development session. Only voluntary faculty within the ALiEM organization were included and there were no exclusion criteria. Groups were limited to 6-8 participants to facilitate small group discussion. Based on the number of recruited participants, two separate groups were formed. One group held its meetings in January of 2017 and the second group met in October of 2017. Because of the innate geographic diversity of volunteer faculty within the ALiEM team, each group was formed of physicians from varying locations across the United States and Canada. While the volunteer faculty at ALiEM come from different specialties and health professions, all of the volunteers for this faculty development program were Emergency Medicine physicians. Demographics of the group are described in Table 1.

**Table 1:** Participant Demographics

Participant Demographics				
Sex	Female 57% (n=8)	Male 43% (n=6)		
Academic Rank	Instructor 14% (n=2)	Assistant Professor 65% (n=9)	Associate Professor 7% (n=1)	Full Professor 14% (n=2)
Geographic Location	US West Coast 29% (n=4)	US Midwest 36% (n=5)	US East Coast 14% (n=2)	Canada 21% (n=3)

Procedures of the Mastermind group

**Figure 1:**
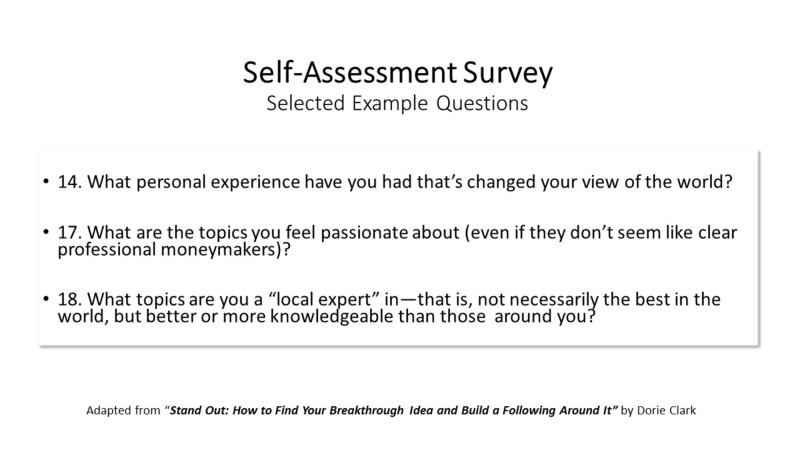
Self-assessment Survey

Each Mastermind group completed two homework assignments and two 90-minute videoconference meetings, using a structured, moderator-facilitated format. Meetings were conducted using participants’ personal computers on Google Hangouts on Air© (Google Inc., Mountain View, CA). As pre-work, participants completed selected questions from a free self-assessment tool created as supplementary, online material for "Stand Out", a book authored by Dorie Clark, adjunct professor at Duke University’s Fuqua School of Business and proponent of the Mastermind group model [23-24]. The self-assessment survey summarized participants professional strengths, weaknesses and current career trajectory. Example questions can be found in Figure 1. Given 6-8 participants per group and the selected questions for each participant to discuss, 90 minutes was thought to be the optimal length of time per session.

In the initial group meeting, the moderator encouraged participants to discuss their self-assessments, current projects, and career challenges. The moderator then facilitated comments from all of the other participants and kept track of time so that all participants could discuss their self-assessments during the 90-minute session. Prior to the second meeting, participants each contributed to a shared, digital document (Google Docs©, Google Inc.) with their suggestions for professional development resources personalized for each of the other participants in the group. The second meeting allowed discussion of these suggested resources, actionable ‘next steps’, and an accountability timeline for each participant. The free, cloud-based platforms and voluntary basis for the Mastermind groups resulted in a zero-cost innovation.

**Figure 2:**
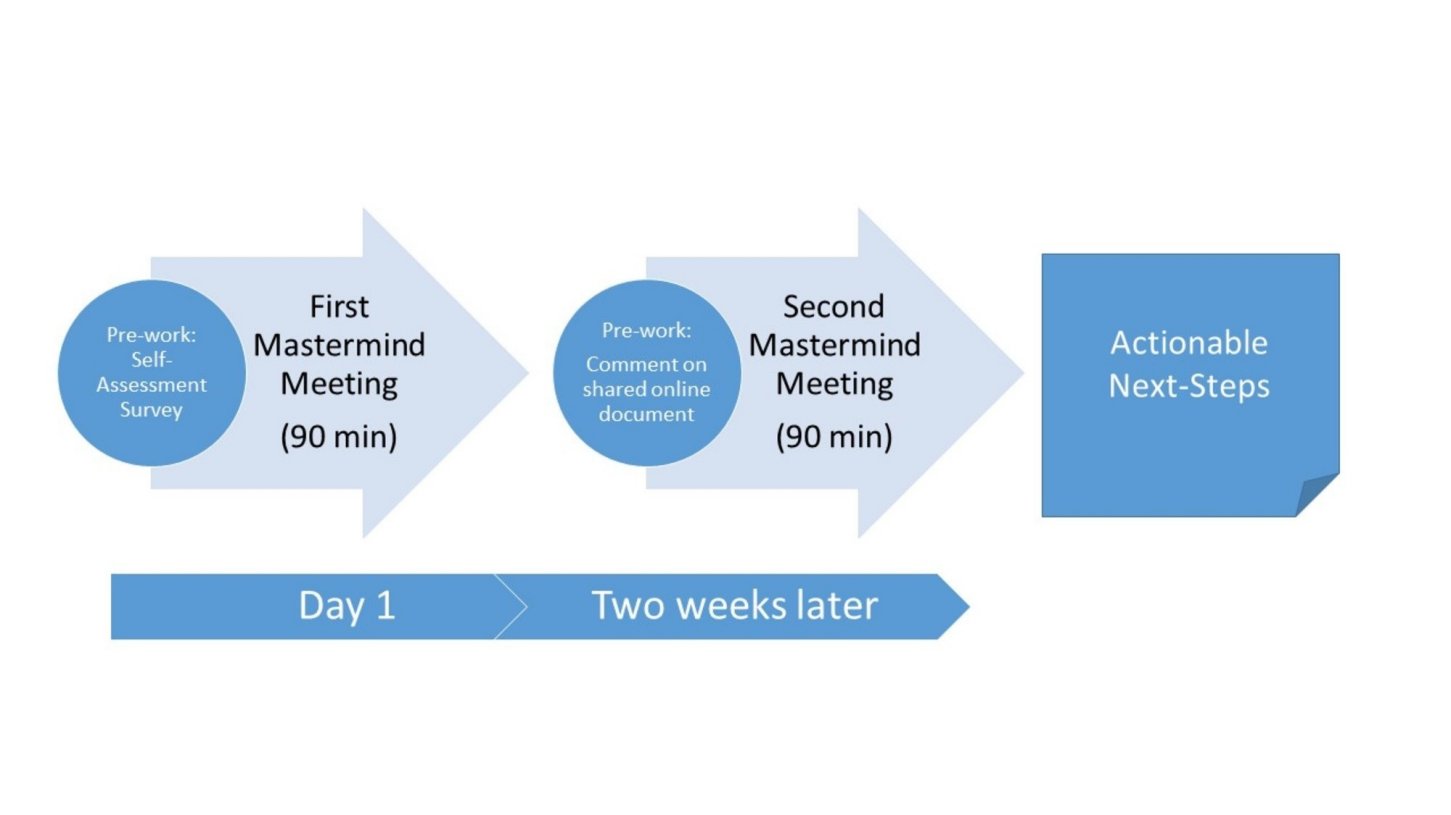
Mastermind Procedures Flowchart 1. Before the first meeting, pre-work self-assessment survey is assigned to all participants. 2. During the first 90-minute of the Mastermind meeting, the participants discuss self-assessments and comment on each other's strengths, weaknesses, and career trajectories. 3. Before the second meeting, each participant contributes to the shared, online document, suggesting resources, connections, and 'next-steps' personalized for each other. 4. The second 90-minute Mastermind meeting is used to discuss action plans for each participant proposed by the group members.

Data collection

In addition to collecting the Mastermind group session notes compiled by the facilitator and participants through the shared, digital document, we also surveyed the participants via anonymous online survey software (SurveyMonkey, San Mateo, CA) to understand participant reactions and obtain program evaluation information.

The post-intervention survey of this program was administered to participants. This survey consisted of three phases: demographic data, participant reactions to the experience, and perceived value. Respondents were asked to rate their Mastermind Group experiences using a Likert Scale ranging from 1 (not valuable, would not recommend to others) to 10 (very valuable, would highly recommend this to others). They were also asked to compare these sessions to prior mentorship experiences using a comparison scale (better than, same as, worse than other mentorship experiences). Finally, qualitative data was gathered about the participants’ experiences via a required free-text box. See Appendix 1 for a listing of all the questions.

Analysis

We describe the demographic composition of the groups, themes discovered in the Mastermind shared digital document notes compiled by the facilitator and participants, and we perform descriptive statistics on our program evaluation survey data.

## Results

Participant demographics

The two groups included two full professors, one associate professor, nine assistant professors, and two instructors, representing 14 different academic medical centers across North America. 57% of participants were female (n=8), and 43% were male (n=6). Compiled summary data of the groups is provided in Table 1.

Description of themes from the Mastermind session notes

A majority of participants received specific resource recommendations during the sessions, including readings (e.g., books, journal articles, blog posts), training courses, or conferences. Many also received introductory email referrals to specific individuals for additional mentorship. This was made possible given the breadth of networks among the participants. All participants had at least one identifiable ‘next step’ related to their reason for participating in the Mastermind group with the goal of being accountable to the group.

Survey results

A post-intervention survey was sent to a convenience sample comprised of the 14 participants, with a final response rate of 100%. The participants rated the Mastermind group experience as 9.4/10 on the Likert scale. When asked to compare Mastermind groups with prior mentorship experiences, 3/14 (21.4%) respondents noted that this was not applicable as this was their first formal mentorship experience, while the remainder of the participants, 11/14 (78.6%) rated these sessions as “much better than prior experiences”, 10/10 on a Likert scale. Participants cited one of two reasons for participating in the Mastermind groups: need for career advice or assistance with a project. Overall, the participants described a synergy of energy, commitment to one another’s longitudinal success, and benefit from the diverse range of talent and expertise in the group as reasons for preferring this model to other models of mentorship. Many of the members discussed plans to replicate this mentorship model in other settings.

Associated costs

Program organizers noted that they used commonly available, free services (Google Hangouts on Air), which allowed them to run this for free. Each individual spent a total of three hours in online discussion forums, as well as the time needed for pre-work completion. The participants and moderators were all uncompensated, volunteer faculty in the ALiEM organization.

## Discussion

Gottlieb and colleagues have previously described the opportunities and barriers to digital mentorship in a recent paper [22]. This application of digital technologies displays that it is acceptable, feasible, and economical to establish digitally-based Mastermind groups between geographically disparate individuals.

Interestingly, the participants did not mention any specific barriers to the Mastermind group, though we acknowledge that at a minimum there is an opportunity cost and minor scheduling challenges inherent in our design. Traditional barriers in mentorship have been previously reported in the literature as: time required for mentorship, lack of academic recognition, lack of financial incentives, “authoritative boss-employee relationship”, lack of availability, and a lack of a good selection of mentors [25]. Our faculty development program overcomes several of these barriers by connecting like-minded individuals across institutions through real-time meetings and connecting a globally distributed team. It also makes it less likely for those at a higher professoriate rank to have any authority or boss-employee relationship with the junior members. By reaching outside of geographic restrictions, the catchment of available mentors was also widened, increasing the number of candidates for whom this program would be relevant.

Limitations

There may be an inherently biased sample of individuals who agreed to participate in our pilot Mastermind program. Many of the individuals that volunteer within the ALiEM organization have a high fluency and affinity for digital technologies. Their experiences, therefore, may not generalize to a population that finds the aforementioned online platforms difficult to navigate. Additionally, volunteers recruited from the ALiEM organization were all from North America and all fluent English speakers. This group of participants may not be a typical cross-section of junior, mid-career, or senior faculty members in terms of their values and preferences around remuneration or academic merit. Finally, volunteers participated in only two, ninety-minute sessions, from which it is difficult to discern any long-term outcome measures or follow-through on proposed ‘next steps’.

## Conclusions

Our experiences suggest that the Mastermind conceptual framework is a feasible, zero-cost, and effective model for professional development for faculty who volunteer their time. This model was easily and cost-effectively replicated from the business literature to a cohort of physicians. Though the model was originally proposed as a method for in-person discussions, we report a more modern, online experience for professional development in our diverse, globally-distributed team.

## Appendices

Survey Questions

1. Which best describes your current professional stage? A. In residency or fellowship training B. 1-5 years out of training C. 5-10 years out of training D. 10+ years out of training

2. What is your academic rank? A. Resident B. Instructor C. Assistant Professor D. Associate Professor E. Full Professor

3. What is your gender? A. Female B. Male C. Prefer not to say

4. What is your age? A. 20-29 B. 30-39 C. 40-49 D. 50-59 E. 60+

5. If you have participated in formal mentorship programs prior to this Mastermind Group, please describe (e.g., mandatory departmental mentorship pairing; "speed-dating" mentorship at a local conference; etc.) (Free Response)

6. Please rate your experience with the Mastermind Group. (1 = NOT Valuable; Would NOT Recommend This To Others; 10 = Very Valuable; Would HIGHLY Recommend This To Others)

7. Please compare this with prior mentorship experiences. A. This program was MUCH WORSE than prior mentorship experiences B. This program was SLIGHTLY WORSE than prior mentorship experiences C. This program was SIMILAR to prior mentorship experiences D. This program was SLIGHTLY BETTER than prior mentorship experiences E. This program was MUCH BETTER than prior mentorship experiences F. Not Applicable

8. When comparing the Mastermind group with prior mentorship experiences (Question #3), why did you feel this way? (Free Response)

9. After this Mastermind Group experience, what is one thing you will do differently? (This may include career, goals, professional development, personal development, or any other aspects you deem important). (Free Response)

10. Did you obtain any resources recommended for you during the discussion (eg. books, articles, online resources)? If so, please describe what you obtained. (Free Response)

11. Have you contacted any of the individuals who were suggested to you? If so, please describe your experience with them. (Free Response)

12. Please describe any other actions that you took as a result of your MasterMind group experience. (Free Response)

## References

[1] Zerzan JT, Hess R, Schur E, Phillips RS, Rigotti N (2009). Making the most of mentors: a guide for mentees. Acad Med.

[2] Schmidt A, Schwedler A, Hahn EG (2010). Does the training of mentors increase the contact frequency and the quality of support in a portfolio-based teaching module?. GMS Z Med Ausbild.

[3] Sambunjak D, Straus SE, Marusic A (2006). Mentoring in academic medicine: a systematic review. JAMA.

[4] Sambunjak D, Straus SE, Marusic A (2010). A systematic review of qualitative research on the meaning and characteristics of mentoring in academic medicine. J Gen Intern Med.

[5] Luckhaupt SE, Chin MH, Mangione CM (2005). Mentorship in academic general internal medicine: results of a survey of mentors. J Gen Intern Med.

[6] Detsky AS, Baerlocher MO (2007). Academic mentoring--how to give it and how to get it. JAMA.

[7] Lindén J, Ohlin M, Brodin EM (2013). Mentorship supervision and learning experience in PhD education. Studies in Higher Education.

[8] Chun JU, Sosik JJ, Yun NY (2012). A longitudinal study of mentor and protégé outcomes in formal mentoring relationships: mentor and protégé outcomes. J Organ Behav.

[9] Fagenson EA (1989). The mentor advantage: perceived career/job experiences of proteges versus non-proteges. J Organ Behav.

[10] Scandura TA (1992). Mentorship and career mobility: an empirical investigation. J Organ Behav.

[11] Morrison LJ, Lorens E, Bandiera G (2014). Impact of a formal mentoring program on academic promotion of Department of Medicine faculty: a comparative study. Med Teach.

[12] Yarris LM, Juve AM, Artino AR Jr (2014). Expertise, time, money, mentoring, and reward: systemic barriers that limit education researcher productivity-proceedings from the AAMC GEA workshop. J Grad Med Educ.

[13] Gusic ME, Zenni EA, Ludwig S, First LR (2010). Strategies to design an effective mentoring program. J Pediatr.

[14] Burlew LD (1991). Multiple mentor model: a conceptual framework. J Career Dev.

[15] Lord JA, Mourtzanos E, McLaren K, Murray SB, Kimmel RJ, Cowley DS (2012). A peer mentoring group for junior clinician educators: four years’ experience. Acad Med.

[16] Cook DA, Bahn RS, Menaker R (2010). Speed mentoring: an innovative method to facilitate mentoring relationships. Med Teach.

[17] Arnold BN, Friedrichsen L, Nishiyama M (2013). Creating your MasterMind: personal and professional development through mastermind groups. Proceedings of the 41st annual ACM SIGUCCS conference on User Services.

[18] Rolfe A (2017). What to look for in a mentor. Korean J Med Educ.

[19] (2013). 7 Reasons To Join A Mastermind Group. Forbes Magazine. October.

[20] Clark D. Create a “Mastermind Group (2018). Create a “Mastermind Group to Help Your Career. Harvard Business Review. August.

[21] Hill N (1937). Think and Grow Rich. http://www.naphill.org/shop/books/think-and-grow-rich/.

[22] Gottlieb M, Fant A, King A (2017). One click away: digital Mentorship in the modern era. Cureus.

[23] Clark D (2015). Stand Out: How to Find Your Breakthrough Idea and Build a Following Around It. https://dorieclark.com/stand-out/.

[24] (2018). Stand Out Self-Assesment. DorieClark.com.

[25] Straus SE, Chatur F, Taylor M (2009). Issues in the mentor-mentee relationship in academic medicine: a qualitative study. Acad Med.

